# Distinct MCM10 Proteasomal Degradation Profiles by Primate Lentiviruses Vpr Proteins

**DOI:** 10.3390/v12010098

**Published:** 2020-01-15

**Authors:** Hao Chang, Lowela Siarot, Ryosuke Matsuura, Chieh-Wen Lo, Hirotaka Sato, Hiroyuki Otsuki, Yoko Aida

**Affiliations:** 1 Viral Infectious Diseases Unit, RIKEN, 2-1 Hirosawa, Wako, Saitama 351-0198, Japan; 2 Laboratory of Viral Infectious Diseases, Department of Computational Biology and Medical Sciences, Graduate School of Frontier Science, The University of Tokyo, 2-1 Hirosawa, Wako, Saitama 351-0198, Japan; 3 Photonics Control Technology Team, RIKEN Center for Advanced Photonics, 2-1 Hirosawa, Wako, Saitama 351-0198, Japan; 4 Laboratory of Global Animal Resource Science, Graduate School of Agricultural and Life Sciences, The University of Tokyo, 2-1 Hirosawa, Wako, Saitama 351-0198, Japan; 5 Nakamura Laboratory, Baton Zone program, Riken Cluster for Science, Technology and Innovation Hub, 2-1 Hirosawa, Wako, Saitama 351-0198, Japan

**Keywords:** primate lentiviruses, Vpr, MCM10, proteasomal degradation, DNA damage response, G_2_/M arrest

## Abstract

Viral protein R (Vpr) is an accessory protein found in various primate lentiviruses, including human immunodeficiency viruses type 1 and 2 (HIV-1 and HIV-2) as well as simian immunodeficiency viruses (SIVs). Vpr modulates many processes during viral lifecycle via interaction with several of cellular targets. Previous studies showed that HIV-1 Vpr strengthened degradation of Mini-chromosome Maintenance Protein10 (MCM10) by manipulating DCAF1-Cul4-E3 ligase in proteasome-dependent pathway. However, whether Vpr from other primate lentiviruses are also associated with MCM10 degradation and the ensuing impact remain unknown. Based on phylogenetic analyses, a panel of primate lentiviruses Vpr/x covering main virus lineages was prepared. Distinct MCM10 degradation profiles were mapped and HIV-1, SIVmus and SIVrcm Vprs induced MCM10 degradation in proteasome-dependent pathway. Colocalization and interaction between MCM10 with these Vprs were also observed. Moreover, MCM10 2-7 interaction region was identified as a determinant region susceptible to degradation. However, MCM10 degradation did not alleviate DNA damage response induced by these Vpr proteins. MCM10 degradation by HIV-1 Vpr proteins was correlated with G_2_/M arrest, while induction of apoptosis and oligomerization formation of Vpr failed to alter MCM10 proteolysis. The current study demonstrated a distinct interplay pattern between primate lentiviruses Vpr proteins and MCM10.

## 1. Introduction

During long co-evolutionary history, viral pathogens keep developing increasingly novel weapons which against hosts to facilitate viral survival and pathogenesis, via encoding not only structural and enzymatic proteins but also various accessory proteins where required. Human immunodeficiency viruses type 1 and 2 (HIV-1 and HIV-2) and other primate lentiviruses encode accessory proteins that enhance viral infectivity [1]. These include Viral protein R (Vpr), Viral infectivity factor (Vif), Viral protein U (Vpu), and negative regulation factor (Nef). In addition, some subsets of primate lentiviruses, including HIV-2 and simian immunodeficiency virus (SIV), also encode a paralog of Vpr termed Viral protein X (Vpx). In general, accessory proteins recruit host proteins that are important for viral replication and/or to antagonize antiviral factors. 

Viral protein Rs (Vprs) are multifunctional accessory proteins, found among all primate lentiviruses, including HIV-1 and HIV-2 and various SIVs. A striking feature of Vpr is its unique potential to promote viral productivity in monocytes/macrophages and in a small population of CD4^+^ T cells [2]. Once encapsulated and released into the cytoplasm, Vpr initiates multiple functions which are consistent with well-organized viral replication circles. Vpr promotes accurate viral genomic RNA reverse transcription [3,4], pre-integration complex (PIC) formation and nuclear localization [5,6,7], transcription regulation of viral and host genes [8], disorders of spliceosome complex processing [9,10], dysregulation of the cell cycle and positive and negative regulation of apoptosis [11,12,13], as well as the activation of DNA damage response pathways [14,15]. Notably, Vpr hijacks the DCAF1 (DDB1)-Cul4-E3 ubiquitin ligase complex resulting in the ubiquitination of numerous cellular targets and ensuing proteasome-dependent degradation [16]. Previous works showed that HIV-1 Vpr interacts with DCAF1 (DDB1)-Cul4-E3 ubiquitin ligase complex to modulate multiple cellular targets’ function and led to proteasome-dependent degradation. Furthermore, such modulation is essential for viral replication and in favor of virus escaping from immune surveillance of hosts [17].

Mini-chromosome maintenance protein 10 (MCM10) is a newly identified cellular target of HIV-1 Vpr, which is recruited to DCAF1-Cul4-E3 ligase for proteasome-dependent degradation [18,19]. As a conserved component of the eukaryotic replisome, MCM10 promotes continuous replication, including initiation of DNA replication, replication fork stability, and DNA damage control [20,21]. The multiple properties of MCM10 are concordant with cell cycle surveillance and thus dysregulation of MCM10 is considered as a common molecular marker of cancer. Previous studies have also suggested that Vpr hijacks DCAF1-Cul4-E3 ligase resulting in MCM10 degradation, which in turn induces G_2_/M arrest [18].

However, it is still unclear whether sequence homology and functional conservation among various primate lentiviruses Vprs are associated with MCM10 proteasome-dependent degradation. Furthermore, how MCM10 degradation regulates ensuing physiological activities is also unrevealed.

The current study firstly investigated whether MCM10 degradation by primate lentiviruses Vprs occurs and whether it was correlated with viral lineages. Accordingly, we synthesized nucleotide sequences of 11 representative primate lentiviruses Vpr/Vpx based on HIV database and performed phylogenetic analyses. Furthermore, distinct MCM10 degradation profiles by primate lentiviruses Vpr/x were mapped through proteasome dependent pathway. Moreover, our findings also indicated that the MCM2-7 interaction region of MCM10 was a determinant domain susceptible to degradation by Vpr. However, MCM10 did not alleviate DNA damage response induced by Vpr proteins. Taken together, the study characterized interaction pattern of MCM10 and various primate lentiviruses Vpr/x through E3 ligase complex hijacking.

## 2. Materials and Methods

### 2.1. Phylogenetic Analysis and Vpr Alleles Preparation

Ninety-six full length HIV/SIV Vpr amino acid sequences were obtained from Los Alamos HIV Database and multiple alignments were performed with MUSCLE algorithm implemented in MEGA 7 [22]. According to alignment results, phylogenetic trees were constructed via neighbor-joining methods with 1000 replicates bootstrap value (cut-off value ≥ 50%). SIVcol Vpr strain was taken as a reference group. In terms of phylogenetic trees, 10 representative HIV/SIV Vpr alleles were chosen as follows: HIV-1 NL4-3 Vpr (accession number, P12520); SIVdeb Vpr (accession number, AAT68805); SIVsyk Vpr (accession number, AAA74709); SIVlst Vpr (accession number, AAF07319); SIVmus Vpr (accession number, ABO61047); SIVmon Vpr (accession number, AAR02379); SIVrcm Vpr (accession number, AAK69677); SIVagm Vpr (accession number, AAA64260); SIVmac Vpr (accession number, AAA47636) and SIVcol Vpr (accession number, AAK01035). HIV-2 Rod10 Vpx (accession number, AYA94987) was selected as the outgroup control for alignment.

### 2.2. Plasmid Construction

In order to generate 11 HIV/SIV Vpr/x expression vectors, HIV-1, SIVdeb, SIVsyk, SIVlst, SIVmus, SIVmon, SIVrcm, SIVagm, SIVmac, and SIVcol, as well as the HIV-2 Vpx, were synthesized (GENEWIZ) according to the nucleotide sequences collected as stated above and subcloned in pcDNA3.1, which contained a N-terminally linked 3 × FLAG tag (pcDNA3.1/3 × FLAG). To generate specific single-site mutant expression vectors of HIV-1 NL4-3 Vpr (accession number, P12520), namely, HIV-1 NL4-3 Vpr K27M, P35A, W54R, C76A, R77Q, and R80A, HIV-1 NL4-3 mutants were introduced into the pME18neo-internal ribosomal entry site (IRES)-ZsGreen1 with FLAG-Vpr (pME18neo/FLAG-Vpr-IRESZsGreen1) using a standard site-directed mutagenesis kit (TAKARA, Shiga, Japan) [23]. To generate N-terminally HA tagged MCM10, cDNA of MCM10 was amplified from total mRNA of HeLa cells and subcloned into pcDNA 3.1 backbone (pcDNA 3.1/HA-MCM10). Domain-deficient mutants of MCM10, namely, HA-1-165, HA-1-427, HA-1-530, and HA-1-655 were also constructed from the pcDNA 3.1/HA-MCM10.

### 2.3. Cell Culture, Transfection, and Drug Treatment

Human embryonic kidney HEK293T cells, HEK293, and Human cervical HeLa cells were maintained in Dulbecco’s Modified Eagle Medium (Gibco, Beijing, China) supplemented with 10% fetal calf serum in a 5% CO_2_ incubator at 37 °C. Plasmid transfection was performed using FuGENE HD (Promega, Madison, WI, USA). For the experiment involving proteasome reversible inhibitor MG132 (Sigma-Aldrich, St. Louis, MO, USA) and irreversible inhibitor Lactacystin (EMD Millipore, Darmstadt, Germany), cells were transfected with the indicated plasmids for 43 h before addition of the inhibitor and cultured for a further 5 h.

### 2.4. Co-Immunoprecipitation Assay

HEK293T cells (4 × 10^6^) were seeded into a 100 mm dish at the day prior to transfection and transiently transfected with either 10 μg of pcDNA3.1/HA-MCM10 or 10 μg of control pcDNA3.1 together with 10 μg of pcDNA3.1/3 × FLAG-Vpr/x. At 48 h of post-transfection, cells were harvested and lysed with NP-40 lysis buffer (50 mM Tris (pH7.5)–150 mM NaCl–0.5% NP-40-1 nM DTT with 1 × protease inhibitors (Roche, Mannheim, Germany)) for 30 min on ice. Next, 100 μg total protein was incubated with 20 μL anti-FLAG M2 affinity gel antibody (Sigma-Aldrich, Saint Louis, MO, USA) in 500 μL binding/wash buffer (10 mM Tris-HCl (pH 7.5)-150 mM NaCl-1% NP-40-1 mM ethylenediamine tetraacetic acid (EDTA)) overnight at 4 °C with rotation (10% mix was kept for input control detection) to purify FLAG labeled proteins in the HEK293T cell lysates. The anti-FLAG M2 antibody affinity gel was collected via centrifugation, washed, and subjected to western blotting with anti-HA mouse monoclonal antibodies (mAb; MBL, Nagoya, Japan) followed by HRP-conjugated goat anti-mouse IgG (Amersham Biosciences, Uppsala, Sweden) for detection of MCM10, of anti-FLAG mouse mAb (MBL, Nagoya, Japan) followed by HRP-conjugated goat anti-mouse IgG (Amersham Biosciences) for detection of Vprs.

### 2.5. Western Blotting

HEK293T cells (2.5 × 10^5^ cells) were seeded into a 12-well-plate at the day prior to transfection, then were transfected with 1 μg of pcDNA 3.1/HA-MCM10 with/without 10 μg of pcDNA3.1/3 × FLAG-lentiviruses Vpr/x. At 48 h following transfection, cells were harvested and lysed for western blotting. Each protein sample was boiled in 4 × SDS sample buffer and electrophoresed via 12% SDS-polyacrylamide gel electrophoresis (PAGE). Proteins were then transferred to a polyvinylidene difluoride membrane (Millipore, Burlington, MA, USA) using a Trans-Blot Turbo apparatus (Bio-Rad, Hercules, CA, USA). The membrane was blocked in 5% skimmed milk in PBST [phosphate-buffered saline (PBS)/0.1% Tween-20] for 1 h and incubated overnight at 4 °C with primary antibodies, such as anti-FLAG rabbit/mouse monoclonal antibodies (mAb; MBL Nagoya, Japan), anti-HA mouse mAb (MBL), anti-α-Tubulin mouse mAb (Sigma-Aldrich, Saint Louis, MO, USA), or anti-MCM10 rabbit polyclonal antibodies (pAb; Proteintech, Posemont, IL, USA), washed with PBST, and incubated with the appropriate secondary antibodies, such as HRP-conjugated goat anti-mouse IgG (Amersham Biosciences, Uppsala, Sweden) or HRP-conjugated goat anti-rabbit-IgG (Amersham Biosciences) at room temperature for 1 h. The chemiluminescent signal was developed using the SuperSignal West Pico chemiluminescent substrate (Thermo Fisher Scientific, Waltham, MA, USA) and imaged using a FluorChem 5500 (Alpha Innotech, San Leandro, CA, USA). Band densities were analyzed by densitometry analysis using ImageJ software (National Institutes of Health, Bethesda, MD, USA).

### 2.6. Immunofluorescence Staining

HeLa cells (2.5 × 10^5^) or HEK293 cells (2.5 × 10^5^) were seeded on cover glasses in a 12-well plate and transfected with 1 μg of pcDNA 3.1/HA-MCM10, with or without 1 μg of pcDNA3.1/3 × FLAG-Vpr. Following 48 h of transfection, immunofluorescence staining was performed as described previously [24]. In brief, cells on a cover slip were fixed with 4% paraformaldehyde for 10 min at room temperature. Paraformaldehyde was then replaced with cold methanol and the cells were maintained at −20 °C for 20 min. The cells were then washed with PBS and incubated with anti-FLAG rabbit mAb (MBL), anti-HA mouse mAb (MBL), or anti-gamma H2AX mouse mAb (Abcam, Cambridge, UK) for 1 h at room temperature. Following further washing with PBS, Alexa Fluor 488 goat anti-rabbit IgG (Invitrogen, Waltham, MA, USA) or Alexa Fluor 594 goat anti-mouse IgG (Invitrogen) was added for 1 h at room temperature in the dark. Nucleus was stained with Hoechst 33342 (Thermo Fisher Scientific) for 5 min in the dark. Coverslips were then rinsed with PBS and mounted on glass slides. Processed samples were visualized using a confocal fluorescence microscope (IX81-FV1000-D/FLUOVIEW System, Olympus, Tokyo, Japan).

### 2.7. Cell Cycle Analysis

HeLa cells (1 × 10^5^) were seeded in a 6-well plate and transfected with 2 μg of pME18neo/FLAG-Vpr-IRESZsGreen1 Vpr wild type or the panel of mutants stated above. After 48 h, the cells were harvested and fixed using 70% ethanol. After being washed twice with PBS, the cells were resuspended in RNase A (Invitrogen; 100 μg/mL) at 37 °C for 20 min and stained with propidium iodide (PI; Sigma; 50 μg/mL) at room temperature for 10 min. Stained cells were analyzed using a BD Accuri^TM^ C6 Plus with a sampler flow cytometer (Becton-Dickinson, Franklin lakes, NJ, USA). The data were analyzed using FlowJo v10 (FlowJo, LLC, Ashland, OR, USA).

### 2.8. Real-Time qRT-PCR Analysis of Human MCM10 mRNA Expression

Total RNA was extracted using the RNeasy mini kit with DNase digestion, according to the manufacturer’s instructions (QIAGEN). RNA was quantified using a NanoDrop spectrophotometer (Thermo Fisher) and stored at −80 °C until use. Reverse transcription was performed using High Capacity RNA-to-cDNA (Thermo Fisher), according to the manufacturer’s manual. qRT-PCR was performed using a Prism 7500FAST sequence detection system (Applied Biosystems). Samples were run in triplicate and all data were normalized to GAPDH mRNA expression as an internal control.

### 2.9. Statistical Analysis

All data were expressed as mean ± standard deviation, based on at least 3 independent experiments. Statistical significance was evaluated using Student’s t-test. Differences were estimated to be significant at *p* < 0.05 (*), and strongly significant at *p* < 0.01 (**) and *p* < 0.001(***).

Correlation efficient was analyzed with linear regression and significance was calculated with Pearson correlation analysis.

## 3. Results

### 3.1. Phylogeny, Multiple Alignments, and Expression of Vpr/x from Representative Strains

**Figure 1:**
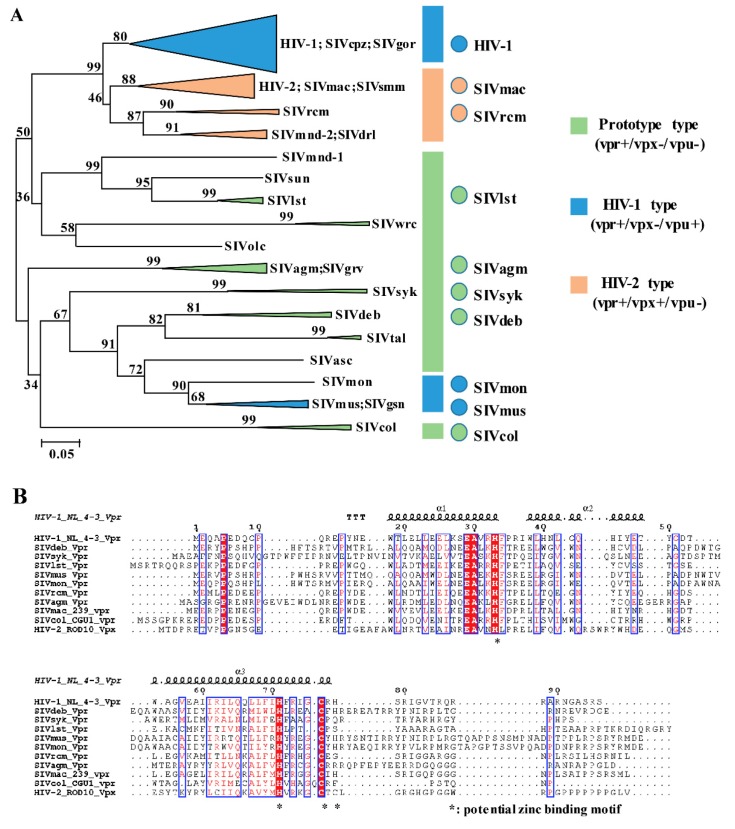

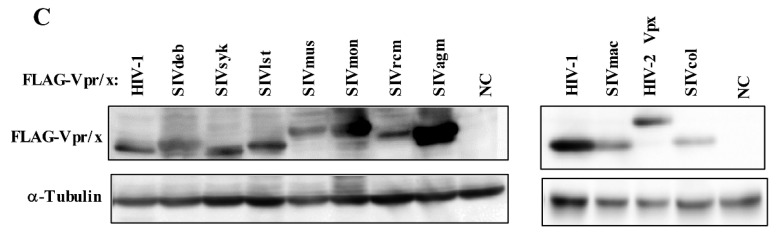
Phylogeny of 96 primate lentiviruses Vprs and multiple alignment and expression of Vpr/x selected from representative strains. (**A**) Phylogenetic tree was constructed from 96 full-length HIV/SIV Vpr amino acid sequences via neighbor-joining methods using 1000 bootstrap replicates. Scale bars depict genetic distance. Representative Vprs from 10 different lineages were selected as later alignment candidates. These originated from viruses belonging to three different groups containing HIV-1 type (HIV-1, SIVmus and SIVmon), prototype (SIVdeb, SIVsyk, SIVlst, SIVagm, and SIVcol) and HIV-2 type (SIVmac, SIVrcm and HIV-2), which are shown in blue, green, and orange, respectively. (**B**) Sequence alignments of candidate HIV/SIV Vprs and HIV-2 Vpx. HIV-1 Vpr was chosen as standard sequence and HIV-2 Vpx as outgroup control. Alignments of HIV/SIV Vpr/x showed sequence and structural conservation, characterized by three α-helices and a potential zinc-binding motif among lentiviruses Vpr/x, indicated by the reference structure, HIV-1 NL4-3, at the top of alignments. (**C**) Expression of 10 HIV/SIV Vprs and 1 HIV-2 Vpx. HEK293T cells were transfected with pcDNA3.1 that encoded 3 × FLAG-tagged HIV/SIV Vpr/x proteins, or the control pcDNA3.1/3 × FLAG (NC: negative control). Transfected cells were harvested at 48 h following transfection and lysates with the equal protein amounts were subjected to western blotting. Positions of Vpr and α-Tubulin are indicated.

In order to cover most HIV/SIV lineages and ensure minimum selection bias, HIV/SIV Vpr proteins derived from 10 lentiviruses strains were chosen using the phylogenetic analysis of 96 full-length Vpr amino acid sequences (Figure 1A). These were as follows: prototype viruses (Vpr+Vpx−Vpu−) were covered by SIVdeb, SIVsyk, SIVlst, SIVagm, and SIVcol Vprs; HIV-1 type viruses (Vpr+Vpx−Vpu+) were covered by HIV-1, SIVmus and SIVmon Vprs; and HIV-2 type viruses (Vpr+Vpx+Vpu−) were covered by SIVmac and SIVrcm.

Structural analysis revealed that full length Vpr forms three amphipathic alpha helices surrounding a hydrophobic core (α-helix 1, 2, and 3) [25,26,27]. It also has a flexible, negatively charged N terminal domain flanking the helices, while its C-terminal domain is also flexible, positively charged, and rich in arginine residues. Based on the phylogeny of primate lentiviruses and virus type classification, amino acid sequences of 10 Vpr proteins from each group were analyzed using multiple alignments via the MEGA 7 program (HIV-2 Rod10 Vpx was added as the external reference). Then structure alignment was re-generated by ESPript 3.0 with HIV-1 Vpr structure as the criterion (Figure 1B). Sequence alignments indicated that all Vpr/Vpx proteins shared conserved tertiary structures. For example, residues framed by a blue-line box depicted similarities in both sequence and structure. Additionally, such similarities were mainly enriched in three α-helices, including the residues 18–34, 38–49, and 54–77. Interestingly, all lentiviral Vpr proteins displayed potential zinc-binding motifs (H33, H71, H76, and *78) located in α-helix 2 and 3, which were similar to the conserved HIV-2 Vpx zinc-binding motif (HHCC). It was suggested that a zinc-binding motif is essential for maintaining both Vpr and Vpx. The potential zinc-binding motif of Vpr/Vpx also engaged E3 ubiquitin ligase complex formation and hijacked it to induce cellular factor degradation [28]. By contrast, flexible C-terminal domains exhibited sequence diversity compared to the central region of Vpr/Vpx proteins. Typically, HIV-2 Vpx was characterized by a poly-proline motif (PPM) in the C-terminal domain (residues from 91 to 97) whereas other lentiviruses Vpr proteins possessed few such properties.

Next, in order to compare the expression and function of these Vpr/x proteins, 10 HIV/SIV Vpr and 1 Vpx were synthesized according to nucleotide sequences collected as stated above. Subsequently, HEK293T cells were transfected with pcDNA3.1 that encoded 3 × FLAG-tagged HIV/SIV Vpr/x proteins, namely, 3 × FLAG-HIV-1, SIVdeb, SIVsyk, SIVlst, SIVmus, SIVmon, SIVrcm, SIVagm, SIVmac, and SIVcol Vprs, and HIV-2 Vpx, or the control, pcDNA3.1/3 × FLAG and examined expression via western blotting with the anti-FLAG-mAb. Specific expression levels of all the 11 HIV/SIV Vpr and Vpx were detectable via western blotting (Figure 1C). We next verified sub-cellular distribution of all the 11 primate Vprs by immunofluorescence staining in Hela cells. For all Vpr proteins, dominant distribution of Vpr in nucleus of HeLa cells was observed [29]. Such results are coherent with previous works.

### 3.2. MCM10 down-Regulation by Primate Lentiviruses Vpr/x Proteins

**Figure 2:**
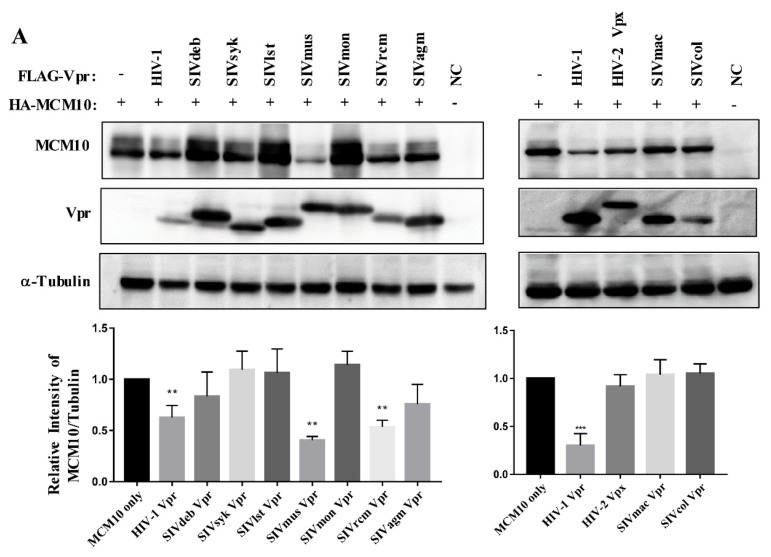

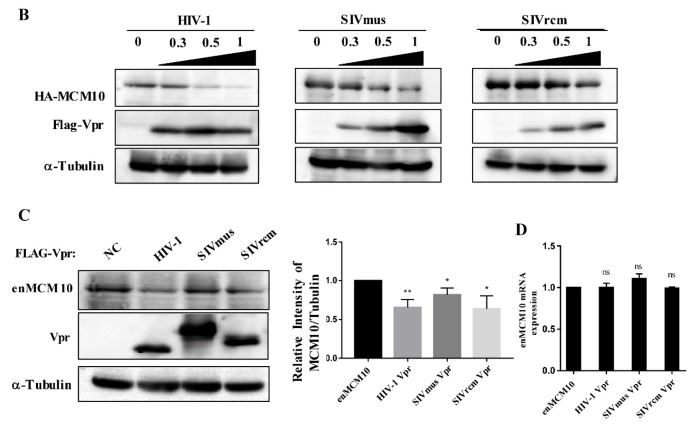
Downregulation of exogenous and endogenous MCM10 by HIV-1, SIVmus, and SIVrcm among 11 primate lentiviruses Vpr/x. (**A**) Downregulation of exogenous MCM10 by 11 primate lentiviruses Vpr/x. HEK293T cells were transiently transfected with pcDNA 3.1/HA-MCM10 together with either pcDNA3.1/3 × FLAG-HIV/SIV Vpr/x, or the control pcDNA3.1/3 × FLAG (NC: negative control). Transfected cells were harvested at 48 h after transfection and lysates with the equal protein amounts were subjected to western blotting with anti-FLAG mouse monoclonal antibodies (mAb), anti-HA mouse mAb, anti-Tubulin mouse mAb (upper panel). The positions of 3 × FLAG-Vpr/x, HA-MCM10 andα-Tubulin are indicated. Band densities of HA-MCM10 and α-Tubulin were quantified by densitometry analysis using ImageJ software. The relative intensities were calculated as the ratio of density of MCM10 to density of α-Tubulin. Each column and error bar represents the mean ± SD of three independent experiments (under panel). (**B**) A dose-dependent manner of downregulation of exogenous MCM10 by HIV-1, SIVmus, and SIVrcm Vprs. HEK293T cells were transiently transfected with pcDNA 3.1/HA-MCM10 together with 0, 0.3, 0.5, or 1.0 µg of either pcDNA3.1/3 × FLAG-HIV-1, SIVmus or SIVrcm Vprs. The positions of 3 × FLAG-Vpr/x, HA-MCM10 and tubulin are indicated (upper panel). (**C**,**D**) Downregulation of endogenous MCM10 protein by HIV-1, SIVmus, and SIVrcm at protein level. HEK293T cells were transiently transfected with either pcDNA3.1/3 × FLAG-HIV-1, SIVmus, or SIVrcm Vprs, or the control pcDNA3.1/3 × FLAG (NC: negative control). After 48 h, endogenous MCM10 protein was examined using western blotting with anti-MCM10 rabbit polyclonal antibodies, anti-FLAG mouse mAb, and anti-Tubulin mouse mAb (**C**), and MCM10 mRNA expression was evaluated using Real-time qRT-PCR analysis (**D**). (**C**) The positions of 3 × FLAG-Vpr, endogenous MCM10 and α-Tubulin are indicated. Band densities of endogenous MCM10 and α-Tubulin were quantified by densitometry analysis using ImageJ software. The relative intensities were calculated as the ratio of density of MCM10 to density of α-Tubulin. Each column and error bar represents mean ± SD for three independent experiments (right panel). The asterisk indicates a statistically significant difference (* *p* < 0.05, ** *p* < 0.01). (**D**) Samples were run in triplicate and all data were normalized to GAPDH mRNA expression as an internal control.

In order to verify the changes in MCM10 expression caused by various HIV/SIV Vpr/Vpx proteins, co-transfection with pcDNA 3.1/HA-MCM10 and pcDNA3.1/3 × FLAG-HIV/SIV Vpr/x was carried out in HEK293T cells and HA-MCM10 expression was monitored via western blotting (Figure 2A). Densities of the HA-MCM10 band were normalized with those of tubulin. The relative density of HA-MCM10 was decreased by co-transfection of HIV-1, SIVmus, and SIVrcm Vpr proteins (Figure 2A lower panel), while other strains failed to induce similar down-regulation of MCM10. MCM10 expression due to the presence of HIV-1 Vpr protein decreased to approximately 62% compared to only-MCM10 control. In addition, MCM10 expression decreased to 40% and 54% due to the presence of SIVmus and SIVrcm Vpr, respectively. In contrast, HIV-2 Vpx did not downregulate MCM10. 

To confirm whether only HIV-1, SIVmus, and SIVrcm Vpr proteins are able to induce MCM10 degradation among 11 primate lentiviruses Vpr/x, HEK293T cells were transiently transfected with 1.0 µg of pcDNA 3.1/HA-MCM10 together with 0, 0.3, 0.5, or 1.0 µg of pcDNA3.1/ 3 × FLAG-HIV/SIV Vpr/x. As predicted, HIV-1, SIVmus, and SIVrcm downregulated MCM10 expression in a dose-dependent manner (Figure 2B), while levels of MCM10 degradation by other Vpr proteins, such as SIVdeb, SIVsyk, SIVlst, SIVmon, SIVagm, SIVmac and SIVcol Vprs, and HIV-2 Vpx, were not effective in each concentration [30]. 

Furthermore, we investigated whether endogenous MCM10 protein expression levels were also susceptible to HIV-1, SIVmus, and SIVrcm Vpr proteins. Likewise, endogenous MCM10 degradation were validated (Figure 2C). MCM10 expression was downregulated to 62%, 79%, and 63% compared to only-MCM10 control, respectively (Figure 2C right panel). Next, we performed real-time qRT-PCR analysis of MCM10 mRNA expression in HEK293T cells, which were transiently transfected with either pcDNA3.1/3 × FLAG-HIV-1, SIVmus, or SIVrcm Vprs and found that MCM10 mRNA expression was detected in similar levels among transfections of HIV-1, SIVmus, and SIVrcm Vpr proteins. This result indicated that down-regulation of endogenous MCM10 by HIV-1, SIVmus, and SIVrcm were induced at protein level (Figure 2D).

Overall MCM10 down-regulation profiles and primate lentiviruses classification were comprehensively analyzed. Interestingly, all Vpr proteins belonging to prototype viruses did not enhance the downregulation of MCM10, while some HIV-1 (HIV-1 Vpr) and HIV-2 (SIVmus and SIVrcm Vpr) type viruses curbed MCM10 expression by varying degrees. However, no functional residues affecting MCM10 down-regulation were found, implying that there may be more than one residue from different sites corporately contributed to decreasing MCM10.

### 3.3. MCM10 Degradation via Proteasome Dependent Pathway

**Figure 3:**
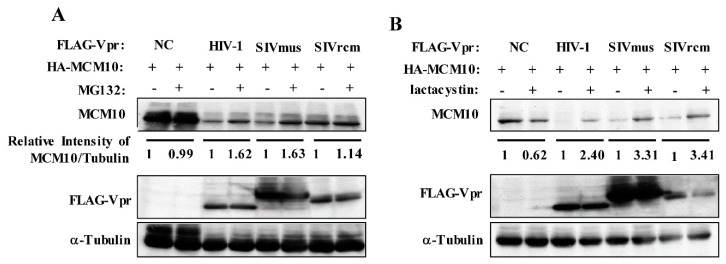
MCM10 degradation by HIV-1, SIVmus, and SIVrcm Vprs via proteasome dependent pathway. HEK293T cells were transiently transfected with pcDNA 3.1/HA-MCM10 together with either pcDNA3.1/3 × FLAG-HIV-1, SIVmus, and SIVrcm Vprs, or the control pcDNA3.1/3 × FLAG (NC: negative control). After 43 h of transfection, cells were treated with 10 μM MG132 (a reversible proteasome inhibitor) or DMSO (**A**), and another irreversible proteasome inhibitor, lactacystin (20 μM) or DMSO (**B**). Cells were harvested at 48 h after transfection and lysates with equal protein amounts were subjected to western blotting with anti-FLAG mouse mAb, anti-HA mouse mAb, anti-α-Tubulin mouse mAb (upper panel). The positions of 3 × FLAG-Vpr, HA-MCM10 and α-Tubulin are shown. Band densities of HA-MCM10 and α-Tubulin were quantified by densitometry analysis using ImageJ software. The relative intensities were calculated as the ratio of density of MCM10 to density of α-Tubulin.

In order to verify whether MCM10 degradation by distinct HIV-1, SIVmus, and SIVrcm Vpr proteins resulted via the proteasomal degradation pathway, MG-132, a reversible proteasome inhibitor, was used to monitor the effects on MCM10 expression. At 43 h following co-transfection of pcDNA3.1/HA-MCM10 together with pcDNA3.1/3 × FLAG-HIV-1, SIVmus, and SIVrcm Vprs or the control pcDNA3.1/3 × FLAG, HEK293T cells were treated with 10 μM MG-132 or DMSO. At 48 h after transfection, HEK293T cells were harvested for western blotting. HIV-1, SIVmus, and SIVrcm Vpr proteins recovered MCM10 expression in MG-132-treated cultures, as compared with that of DMSO-treated cultures (Figure 3A). By contrast, MG-132 exerted no effect on MCM10 expression in cells transfected with only the control, pcDNA3.1/3 × FLAG. This result was confirmed with another irreversible proteasome inhibitor, lactacystin, which also targets the 20S proteasome resulting in proteasomal degradation inhibition. Treatment with lactacystin (20 μM) resulted in an increase in MCM10 expression that was higher than the increase caused by MG 132 (Figure 3B), indicating that lactacystin is an irreversible inhibitor and its activity is more specific as compared with reversible inhibitor MG132, and thus the effect of MG132 may be smaller than that of lactacystin.

### 3.4. Interaction between MCM10 and HIV-1, SIVmus, and SIVrcm Vprs

**Figure 4:**
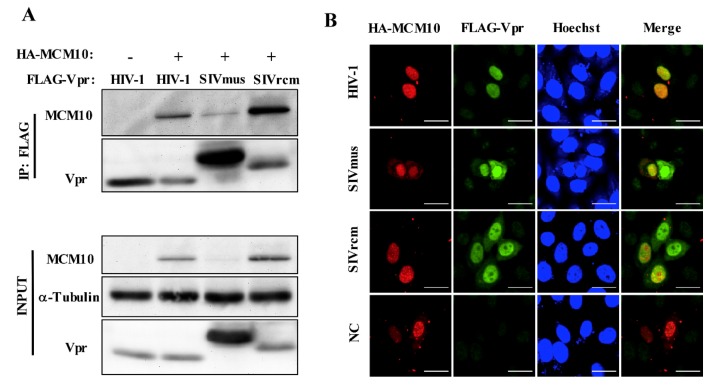
MCM10 interacts and colocalizes with HIV-1, SIVmus, and SIVrcm Vprs. (**A**) HEK293T cells transiently transfected with either pcDNA 3.1/HA-MCM10, or the control, pcDNA 3.1, together with either pcDNA3.1/3 × FLAG-HIV-1, SIVmus, or SIVrcm Vprs. At 48 h following transfection, cell lysates were collected and incubated overnight with anti-FLAG agarose beads. Subsequently, cell lysate inputs and agarose beads were collected, washed, and subjected to western blotting with anti-HA mouse mAb followed by HRP-conjugated goat anti-mouse IgG for detection of MCM10, of anti-FLAG mouse mAb followed by HRP-conjugated goat anti-mouse IgG for detection of Vprs. The positions of 3 × FLAG-Vpr, HA-MCM10 and α-Tubulin are indicated. (**B**) HeLa cells on the cover glass were transiently transfected with pcDNA 3.1/HA-MCM10 together with either pcDNA3.1/3 × FLAG-HIV-1, SIVmus and SIVrcm Vprs, or the control, pcDNA3.1/3 × FLAG. At 48 h following transfection, cells were stained with anti-FLAG rabbit mAb followed by Alexa Fluor 488 goat anti-rabbit IgG to detect Vpr (green), with anti-HA mouse mAb followed by Alexa Fluor 594 goat anti-mouse IgG to detect MCM10 (red), and with Hoechst 33342 to detect nucleus (blue) and observed using a FV-1000 fluorescence microscope. Merged images (orange) indicate localization pattern of both proteins. Bar = 20 µm.

To investigate the interaction between MCM10 and Vprs, including HIV-1, SIVmus and SIVrcm Vprs, we performed co-immunoprecipitation using anti-FLAG beads in HEK293T cells transfected with pcDNA3.1/HA-MCM10, together with either pcDNA3.1/3 × FLAG-HIV-1, SIVmus, or SIVrcm Vprs (Figure 4A). All 3 × FLAG tagged Vpr proteins were immunoprecipitated by HA-MCM10. By contrast, no specific HA-MCM10 band was detected in cells transfected with the control pcDNA3.1/ 3 × FLAG. 

To confirm this interaction, we performed an immunofluorescence assay in HeLa cells. HeLa cells were transiently transfected with pcDNA 3.1/HA-MCM10 together with either pcDNA3.1/3 × FLAG-HIV-1, SIVmus and SIVrcm Vprs, or the control pcDNA3.1/3 × FLAG, and cells were stained with anti-FLAG mAb followed by Alexa Fluor 488 goat anti-rabbit IgG to detect Vpr (green), with anti-HA mAb followed by Alexa Fluor 594 goat anti-mouse IgG to detect MCM10 (red) and with Hoechst 33,342 to detect nucleus (blue) (Figure 4B). HA-MCM10 was predominantly concentrated in the nucleus as a form of discrete replication foci. HIV-1, SIVmus, and SIVrcm Vpr expression also accumulated mostly in nucleus of HeLa cells. On the other hand, Vpr distribution was observed to aggregate in a dispersed form around the nucleoli. Vpr proteins partially colocalized with the MCM10 foci in the nucleus in merged images (orange) (Figure 4B).

### 3.5. MCM 2-7 Interaction Region of MCM10 Susceptible to Degradation by Vprs

**Figure 5:**
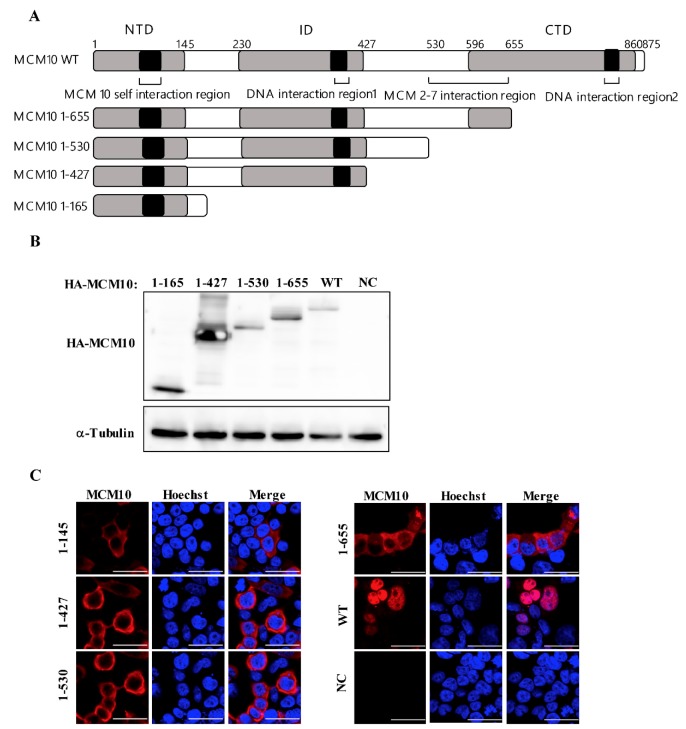
Expression and localization of MCM10 mutants. (**A**) Schematic diagram shows the domain structures of wildtype and MCM10 truncation mutants. Positions of the predicted N-terminal domain (NTD, 1-165), internal domain (ID, 230-427), C-terminal domain (CTD, 596-860), recently identified MCM2-7 interaction region (530-655). MCM10 self-interaction region involved in NTD and 2 DNA interaction regions located in ID and CTD are indicated. (**B**) Expression of MCM 10 mutants. HEK293T cells were transiently transfected with pcDNA3.1/HA-MCM10 WT, 1-165, 1-427, 1-530, or 1-655, respectively. Transfected cells were harvested at 48 h of post-transfection and lysates with the equal protein amounts were subjected to western blotting. The positions of HA-MCM10 and α-Tubulin are indicated. (**C**) Subcellular distribution of MCM 10 mutants. HEK293 cells were transiently transfected with either pcDNA3.1/MCM10 HA-WT, HA-1-165, HA-1-427, HA-1-530, or HA-1-655 and the subcellular distribution of MCM 10 mutants was determined via immunofluorescence staining with anti-HA mouse mAb at 48 h post-transfection. The nucleus was stained with Hoechst 33342 and observed using an FV-1000 fluorescence microscope. Bar = 20 µm.

MCM10 is composed of different domains as follows: N-terminal domain (NTD, amino acids 1-145) responsible for MCM10 self-oligomerization, internal domains (ID, amino acids 230-427) interacting with proliferating cell nuclear antigen (PCNA) and DNA polymerase-α (Pol-α), C-terminal domain (CTD, amino acids 596-860) that interacts with DNA and polymerase-α, and MCM 2-7 interaction region (amino acids 530-655) mediating MCM10 interaction with MCM 2-7 complex, which is also essential for MCM10 nuclear localization (Figure 5A) [21]. To investigate the determinant domain of MCM10 susceptible to degradation by Vpr proteins, pcDNA3.1 expression vectors encoding HA tagged domain-deficient mutants of MCM10, namely, HA-1-165, 1-427, 1-530, and 1-655 were constructed (Figure 5A) and transfected into HEK293T cells. Then, the expression and distribution of these were examined by western blotting and immunofluorescence staining, respectively (Figure 5B,C). The expression of all domain-deficient mutants of MCM10 was specifically detectable via western blotting using anti-HA mAb (Figure 5B). Wildtype MCM10 aggregated in the nucleus and formed typical replication foci (Figure 5C). By contrast, MCM10 (1-655) distribution detected in the cytoplasm was possibly caused by a lack of unidentified nuclear localization signals (NLSs) in CTD. Besides, a small fraction of MCM10 (1-655) was still localized in the nucleus but typical foci formation disappeared. Mutant MCM10 (1-530), MCM10 (1-427), and MCM10 (1-165) were localized in the cytoplasm. 

**Figure 6:**
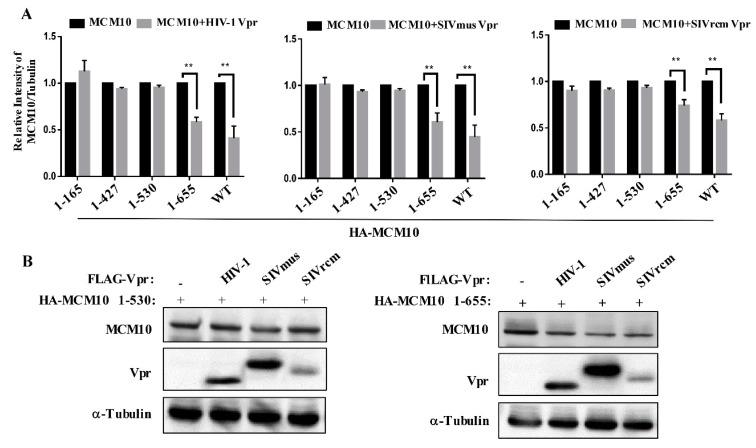
MCM 2-7 interaction region is susceptible to degradation by HIV-1, SIVmus, and SIVrcm Vprs. (**A**) HEK293T cells were transiently transfected with either pcDNA3.1/HA-MCM10 WT, HA-1-165, HA-1-427, HA-1-530, or HA-1-655 together with either pcDNA3.1/3 x FLAG-HIV-1, SIVmus, or SIVrcm Vprs. The cells were then harvested at 48 h after transfection and lysates with equal protein amounts were subjected to western blotting. Band densities of HA-MCM10 and α-Tubulin were quantified by densitometry analysis using ImageJ software. The relative intensities were calculated as the ratio of density of MCM10 to density of α-Tubulin (lower panel). Each column and error bar represents the mean ± SD for three independent experiments (right panel). The asterisks indicate a statistically significant difference (** *p* < 0.01). (**B**) HEK293T cells were transiently transfected with either pcDNA3.1/HA MCM10-1-530, or 1-655 together with either pcDNA3.1/3 × FLAG-HIV-1, SIVmus, or SIVrcm Vprs to analyze western blotting. The positions of 3 × FLAG-Vpr, HA-MCM10 and α-Tubulin are indicated.

Next, we investigated the determinant domain of MCM10 degradation by HIV-1, SIVmus, and SIVrcm Vpr. HEK293T cells were transiently transfected with either pcDNA3.1/HA-MCM10 WT, 1-165, 1-427, 1-530, or 1-655 together with either pcDNA3.1/3 × FLAG-HIV-1, SIVmus or SIVrcm Vprs and the expression of each HA-MCM10 mutant was monitored via western blotting, wherein the densities of each band were normalized with those of tubulin (Figure 6A). The MCM10 mutant, 1-655, missing most parts of CTD but still maintaining the MCM2-7 interaction region, was still susceptible to degradation by HIV-1, SIVmus, and SIVrcm Vpr. This suggested that the CTD of MCM10 expression may not be affected by being engaged by Vprs. By contrast, the MCM mutant, 1-530, missing both MCM 2-7 interaction and CTD domains, was resistant to degradation by Vpr mediation. Moreover, the truncation mutants, 1-427 and 1-165, were both resistant to downregulation by Vprs. Collectively, the region 530-655 appears to be a key region that is responsible for MCM10 proteolysis by Vpr.

In order to confirm above results, MCM10 1-655 or 1-530 was co-transfected with 3 Vprs, following which coherent results were obtained via western blotting (Figure 6B), where MCM10 1-655 retained susceptibility to Vprs, while MCM 1-530 showed resistance to degradation by Vprs.

### 3.6. MCM10 Failure to Alleviate DDR Inducted by Primate Lentiviruses Vprs 

**Figure 7:**
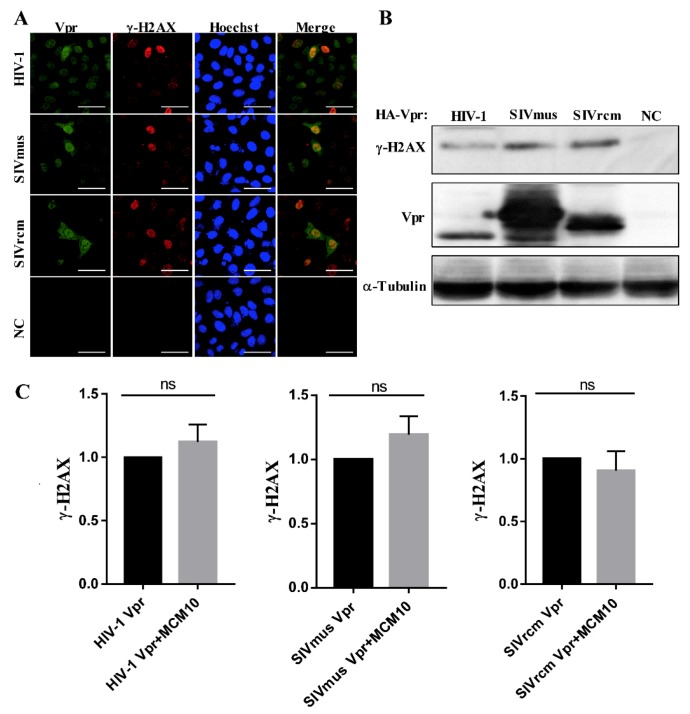
MCM10 did not alleviate DNA damage response (DDR) induced by HIV-1, SIVmus, and SIVrcm Vpr proteins. (**A**,**B**) HEK293T cells were transiently transfected with either pcDNA3.1/3 x FLAG-HIV-1, SIVmus and SIVrcm Vprs, or the control pcDNA3.1/3 × FLAG (NC: negative control). (**A**) Transfected cells were stained with anti-FLAG rabbit mAb followed by Alexa Fluor 488 goat anti-rabbit IgG to detect Vpr (green) and anti-γ-H2AX mouse mAb followed by Alexa Fluor 594 goat anti-mouse IgG to detect γ-H2AX (red) at 48 h of post-transfection, and the nucleus was stained with Hoechst 33,342 and observed using an FV-1000 fluorescence microscope. Bar = 20 µm. (**B**) Transfected cells were harvested at 48 h of post-transfection and lysates with equal protein amounts were subjected to western blotting. The positions of 3 × FLAG-Vpr, γ-H2AX and α-Tubulin are indicated. (**C**) HEK293cells were transiently transfected with pcDNA 3.1/HA-MCM10 together with either pcDNA3.1/3 × FLAG-HIV-1, SIVmus and SIVrcm Vprs, or the control, pcDNA3.1/3 × FLAG. Transfected cells were harvested at 48 h of post-transfection and lysates with equal protein amounts were subjected to western blotting. Densities of γ-H2AX were normalized with those of tubulin. Each column and error bar represents the mean ± SD for three independent experiments.

Previous studies showed that MCM10 prevented DNA damage during the replication process [20]. The speculation that DNA damage response (DDR) may be involved in the degradation process led to the need to explore possible consequences of MCM10 degradation by Vprs. Variant histone H2AX (H2AX), a DDR marker, is phosphorylated (γ-H2AX) in response to DNA double strand breaks (DSBs) by intra- or inter-pathogens or other environmental irritants. Firstly, we investigated whether these 3 Vprs provoked a DNA damage response. HEK293 cells were transiently transfected with either pcDNA3.1/3 × FLAG-HIV-1, SIVmus and SIVrcm Vprs, or the control pcDNA3.1/3 × FLAG, following which transfected cells were examined for γ-H2AX foci formation, using immunofluorescence staining with anti-γ-H2AX mAb. As shown, HIV-1, SIVmus, and SIVrcm Vpr induced γ-H2AX foci to aggregate in the nucleus of HEK293 cells (Figure 7A). By contrast, HEK293 cells transfected with the control vector was negative for immunofluorescence of γ-H2AX. Similarly, γ-H2AX expression in all three groups of HEK293T cells transfected with either pcDNA3.1/3 × FLAG-HIV-1, SIVmus, or SIVrcm Vprs was increased, while γ-H2AX expression in cells transfected with the control pcDNA3.1/3 × FLAG vector was not (Figure 7B). 

To clarify whether MCM10 alleviates DNA damage induced by the 3 Vprs, HEK293T cells were co-transfected with pcDNA 3.1/HA-MCM10 and either pcDNA3.1/3 × FLAG-HIV-1, SIVmus, or SIVrcm Vprs, and γ-H2AX expression was monitored via western blotting with anti-γ-H2AX mouse mAb and anti-Tubulin mAb, wherein band densities of γ-H2AX were normalized using those of tubulin (Figure 7C). Interestingly, γ-H2AX expression levels increased by the 3 Vprs remained unaltered following the addition of MCM10. The relative intensity of γ-H2AX expression showed no significant differences with or without MCM10.

### 3.7. Correlation of MCM10 Degradation with HIV-1 Vpr G_2_/M Arrest 

**Figure 8:**
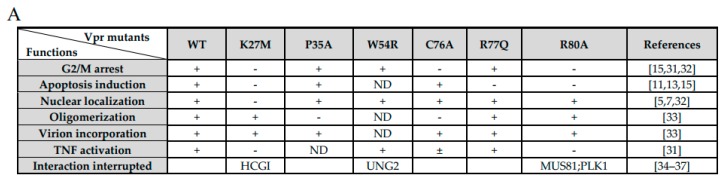

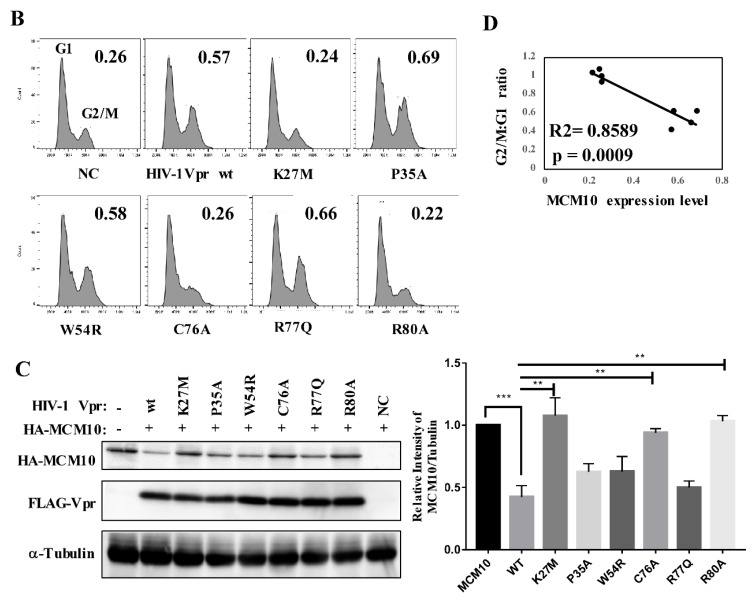
MCM10 degradation by HIV-1Vpr is positively correlated with G_2_/M arrest function of HIV-1 Vpr. (**A**) The table summarizes previous determined functional properties of various HIV-1 Vpr mutants. Sources: [5,7,11,13,15,31,32,33,34,35,36,37]. (**B**) HEK293 cells were transfected with pME18neo/FLAG-IRESZsGreen1 that encoded FLAG-tagged HIV-1 Vpr wild type and a panel of mutants stated above. At 48 h after transfection, cells were harvested to analyze DNA content and stained with propidium iodide. ZsGreen1-positive cells were analyzed using a BD Accuri^TM^ C6 Plus with a sampler flow cytometer. For each mutant, 10000 events were acquired and subsequent G_2_/M:G_1_ ratio was calculated using FlowJo software. (**C**) HEK293T cells were transiently transfected with either pcDNA3.1/HA-MCM10 together with either HIV-1 pME18neo FLAG-tagged HIV-1 Vpr, wild type and a panel of mutants. Transfected cells were harvested at 48 h after transfection and lysates with the equal protein amounts were subjected to western blotting (left panel). The positions of 3 × FLAG-Vpr, MCM10 and α-Tubulin are indicated. Band densities of HA-MCM10 and α-Tubulin were analyzed by densitometry analysis using ImageJ software (right panel). The relative intensities were calculated as the ratio of density of MCM10 to density of α-Tubulin. Each column and error bar represents the mean ± SD for three independent experiments. The asterisks indicate a statistically significant differences (** *p* < 0.01, *** *p* < 0.001). (**D**) Correlation between MCM10 degradation and G_2_/M arrest by HIV-1 Vpr mutants. The line represents the approximate curve. *R* = Pearson’s correlation coefficient (*p* = 0.0009).

Previously, HIV-1 Vpr was found to enhance its G_2_/M arrest effect by increasing MCM10 degradation via proteasome-dependent pathway [18]. However, considering multiple functions of HIV-1 Vpr to cellular targets, a question arose as to whether other functions of Vpr also played roles. Firstly, seven typical HIV-1 Vpr mutants were summed up and characterized (Figure 8A). HIV-1 Vpr K27M, C76A, and R80A curbed the cell cycle at the G_2_/M phage [15,31,32]; K27M, R77Q, and R80A impaired the function of apoptosis induction [15]; P35A specifically lacked Vpr oligomerization [33]; and W54R failed to interact with the host factor UNG2 [34]. Secondly, we generated the expression vectors pME18neo/FLAG-Vpr-IRESZsGreen1 encoding the HIV-1 Vpr mutants, K27M, P35A, W54R, C76A, R77Q, and R80A, and transfected HEK293T cells for cell cycle analysis. The expression of all Vpr mutants were confirmed by western blotting. Three mutants, K27M, C76A, and R80A, decreased cell cycle arrest activity at the G_2_/M phase compared with wildtype HIV-1 (Figure 8B), indicating the three mutants failed to induce cell cycle blocking.

Next, we performed co-transfection with pcDNA 3.1/HA-MCM10 and pME18neo/FLAG-Vpr, encoding HIV-1 Vpr mutants, in HEK293T cells and monitored HA-MCM10 expression via western blotting (Figure 8C, left panel). Densities of the HA-MCM10 band were normalized with those of tubulin. The expression of HIV-1 mutants, K27M, C76A, and R80A reverse MCM10 degradation, compared with that of the HIV-1 wildtype (Figure 8C, right panel). By contrast, P35A, W54R, and R77Q failed to reverse MCM10 proteasome-dependent degradation. 

Finally, quantitative data related to MCM10 degradation profiles and G_2_/M:G_1_ ratios, associated with Vpr mutants, indicated a high correlation (R^2^ = 0.8589; *p* = 0.0009) with each of the functions tested (Figure 8D). The G_2_/M arrest function of HIV-1 Vpr was specifically correlated with MCM10 degradation. By contrast, R77Q, an apoptosis induction-deficient mutant, downregulated MCM10 expression, demonstrating that MCM10 expression levels were not affected by the apoptosis function of HIV-1 Vpr. Another mutant P35A, which lost the Vpr oligomerization function, also failed to reverse MCM10 degradation.

## 4. Discussion

Previous studies have indicated that HIV-1 Vpr increased MCM10 degradation by manipulating DCAF1-Cul4-E3 ubiquitin ligase for proteasome dependent degradation pathway and that such degradation was related to Vpr-mediated G2/M arrest. However, it was unclear whether various primate lentiviruses Vprs also complied with MCM10 proteasome dependent degradation. The current study reached three major conclusions regarding MCM10 degradation pattern by various primate lentiviruses Vpr proteins. Firstly, the study revealed that MCM10 degradation resulting from identical proteasome pathways was caused by distinct SIVmus and SIVrcm Vprs, in addition to HIV-1 Vpr. However, Vpr proteins derived from prototype virus lineages lost the ability to degrade MCM10, implying that MCM10 degradation is associated with species specificity of Vprs. Secondly, our results demonstrated that MCM10 interacted with HIV-1, SIVmus, and SIVrcm Vprs. Furthermore, the MCM2-7 interaction region of MCM10 was the determinant region susceptible to degradation by these 3 Vprs. Thirdly, our data showed that although the γ-H2AX expression levels increased, the 3 Vprs remained unaltered following overexpression of MCM10 in 293T cells, suggesting that MCM10 did not alleviate the DNA damage response induced by the 3 Vprs.

In this study, according to phylogenetic outcomes, 10 representative Vpr proteins from different primate lentiviruses lineages were selected and synthesized. Interestingly, a potential zinc-binding motif (H33, H71, H76, and *78) [38] was found among α-helices 2 and 3 in the Vpr proteins from diverse virus strains. Multiple zinc-binding regions involved in viral proteins are indispensable for negotiating with host factors. For instance, the zinc-binding region (HX_5_CX_17-18_CX_3-5_H) of HIV-1 Vif mediated interaction with Cul5 E3 ligase to exert ubiquitination targeting APOBEC3G [39,40]. Two other zinc-binding sites in the nucleocapsid (NC) of HIV-1 also play an important role in the interaction with nucleus acids of PSI RNA and the eventual promotion of HIV-1 genomic RNA packaging into virus particles [41,42]. However, there is little evidence indicating whether such Vpr sequences mimic the full role played by typical HHCC zinc-binding motifs. Recently, some studies have revealed that the HHCH motif of HIV-1 Vpr, which is positioned parallel to that of HIV-2 Vpx, may show capacity to interact with the E3 ligase complex [28]. However, whether the potential zinc-binding motif contributes to the function of Vprs remains unclear.

Among Vprs from 10 lineages, HIV-1, SIVmus, and SIVrcm Vprs were identified as having varying capacity to specifically curb the expression of MCM10, while other strains failed to do so. Furthermore, MCM10 was colocalized with such Vprs in the nucleus and formed complexes with them. Interestingly, it is suggested that Vprs originating from prototype viruses are unable to induce downregulation of MCM10. Multiple alignment results indicated that there were no distinguishable sequences or point features that would enable differentiation of the capacity to degrade MCM10. Accordingly, it is speculated that more than one amino acid, or a combination of amino acids, in distinct virus strains may perform the function of MCM10 degradation. However, more proof is required to determine whether Vpr proteins of whole prototype virus lineages exhibit MCM10 degradation properties. 

The region encompassing amino acids 530-655, also known as the MCM2-7 interaction region, was mapped as a determinant domain of MCM10 degradation under induction by Vpr [20,43]. Previously, little was known about the 530-633 region of MCM10, identified as a newly identified functional domain, flanked by an ID and involved in parts of CTD. This region exhibits little sequence characterization or secondary structures, by way of sequence alignments and structure prediction, in spite of the compositional bias of hydrophobic amino acids [44,45]. This flexible region is also partly responsible for the intrinsically disordered proteins (IDP). Some studies revealed that IDPs adopted multiple structures and were inclined to enfold, thus mediating binding with other targets of interest [46]. 

Once released into target cells, a virion-associated HIV-1 Vpr initiates multiple functions to facilitate its replication. MCM10 degradation by HIV-1 Vpr was found to induce G_2_/M cell arrest in Hela cells [18]. However, whether Vpr played other roles in MCM10 degradation remains unknown. Therefore, we constructed a series of functionally deficient Vpr mutants and performed MCM10 degradation profiling. Our mutagenesis assay indicated that the G_2_/M cell cycle, instead of apoptosis induction, oligomerization, or nuclear localization, was correlated with MCM10 expression. This finding supported the key role played by MCM10 in cell cycle modulation.

Diverse viruses negotiate with and eliminate cellular factors, by exploiting host metabolism pathways to facilitate virus replication and escape host immune surveillance. Particularly, the ubiquitin dependent degradation pathway is one of the attractive machineries manipulated by multiple accessory proteins, such as, Vif, Vpu, and Vpx, of primate lentiviruses. Predominantly, Vpr was found to invoke increasingly numerous host factors for proteasome dependent degradation, such as MCM10, MUS81, helicase-like transcription factor (HLTF), Exonuclease 1 (Exo1), and histone deacetylases (HDACs) [35,36,37,47,48]. Interestingly, these cellular targets also respond to DNA damage from viruses or other environmental stimulants. For instance, HLTF labels the proliferating cell nuclear antigen (PCNA) with Lys-63 polyubiquitin chain to reverse leading strand replication with that of the lagging strand, a rather undamaged template at replication level error correction. Exo1, another target of HIV-1 Vpr, was also depleted via the proteasomal degradation pathway. Vpr may possibly load Exo1 onto the E3 ligase complex and remodel the post-replication DNA repair machinery independently of PCNA bridging. However, the correlation between Exo1 depletion by Vpr and DNA damage response remains unknown [48,49]. 

DNA damage and late S/G2 phase arrest are induced through MCM10 siRNA treated cells. During replication, MCM10 depletion supposedly blocks the synthesis of the lagging DNA strand, and the subsequent replication fork stalling also generates phosphorylated H2AX. The DSB signal cascade eventually leads to cell cycle arrest [50]. In addition, HIV-1 was found to enhance MCM10 degradation which invoked G_2_/M cell cycle blocking [18]. Mutagenesis and cell cycle analysis also revealed that the degree of MCM10 degradation was correlated with cell cycle arrest with HIV-1 Vpr. However, in our study, MCM10 alone does not alleviate the DNA damage induced by 3 Vpr proteins, suggesting complexity of DNA modulation exposing to viral pathogens. It is still unclear whether the primary role of Vpr on DNA damage exceeds resultant DNA repair machinery activation to favor the purpose of virus replication. Taken together, the results of our studies highlight distinct interplay model of host factor MCM10 with various primate lentiviruses Vpr proteins and their ensuing roles on physiological alternation of cellular targets partly. This research presents a model of primate lentiviruses Vprs antagonism against increasingly found host factors and “arm-race” of host-virus coevolution. 

## 5. Conclusions

This study revealed that distinct MCM10 degradation profiles by primate lentiviruses Vpr/Vpx proteins through proteasome degradation pathway. Particularly, HIV-1, SVImus and SIVrcm Vpr curbed MCM10 expression, while Vpr derived from other 8 Vpr/Vpx did not. Interestingly, colocalization and interaction of MCM10 and these three Vpr proteins also were observed. And MCM2-7 interaction region was susceptible to degradation through proteasomal degradation pathway. For HIV-1, G2/M interruption was directly related with MCM10 degradation but other Vpr function defects were not. However, further experiment is need to investigate whether such set of DNA damage response proteins exert synergistic roles to interact with accessory protein Vpr.
